# There is no horizontal gravity force in geopotential coordinates

**DOI:** 10.1073/pnas.2416636121

**Published:** 2024-11-18

**Authors:** James C. McWilliams

**Affiliations:** ^a^Department of Atmopsheric and Oceanic Sciences, University of California, Los Angeles, CA 90095

**Keywords:** gravity force, Earth’s rotation, geopotential coordinates

## Abstract

In response to a recent challenge to the longstanding practice in modeling large-scale circulations in the atmosphere and ocean that neglects any horizontal component of Earth’s gravity-rotation force, this paper demonstrates that a coordinate transformation into geopotential coordinates has no such horizontal force. This framework should be understood as the justification for and, if warranted, the basis for making further refinements to such geophysical models.

## Significance

Earth’s rotation and gravity field provide an important force in geophysical fluids. The gravity field in particular has a modest degree of spatial complexity, hence so does its force. However, by transforming the momentum equation into a coordinate system with one axis locally aligned with this force, called the vertical direction, there is then no force component in the horizontal directions. This mathematical result contradicts some recently published contrary assertions, and it provides a mathematical demonstration in support of a wide-spread historical understanding on this matter.

Earth’s gravity field is nearly spherically symmetric (or better, spheroidally), and Earth’s rotation rate is mostly constant, even though neither is strictly true. As a consequence, gravity is mostly considered as vertically aligned, and a “traditional approximation” is made for the Coriolis force that only considers the projection of the rotation vector in the vertical direction. For brevity, we use the term gravity to denote both the gravitational attraction to Earth’s mass and the centrifugal force of Earth’s rotation about its polar axis.

It has long and widely been understood in geophysical fluid dynamics that the meaning of vertical is parallel to Earth’s gravitational-centrifugal potential force [e.g., ref. 1, section 213: “When in relative equilibrium, the (ocean’s) free surface is of course a level-surface with respect to gravity and centrifugal force”], although the common practice in both research and teaching has been to write the dynamical equations as if the gravity-rotation field were spatially uniform over the atmospheric or oceanic domains of interest. To my knowledge, no one has challenged this practice until a recent dispute over the advocation of “horizontal gravity” (2–5) and its refutations (e.g., refs. 6–8). Because this dispute remains unresolved in the published literature, this note is an explicit demonstration of the statement in the title.

Here, we consider corrections to these simple representations with uniform gravity. We do so as a proof of concept by considering a spatially variable geopotential function Φ that provides a force ∇Φ, but all in an “absolute” Cartesian coordinate framework, rather than a more realistic spheroidal one. The generalization to a proper planetary geometry should be straightforward, if lengthy (n.b., Chaps. 7, 8, and 12 in ref. 9); e.g., an assessment of the errors in approximating fluid equations in a spheroidal geometry with an spherical one is in ref. 10.

## 1. Geopotential Coordinates

Consider “absolute” Cartesian coordinates (ξ,η,ζ,τ) with Φ(ξ,η,ζ); the extremely small time derivatives of Earth’s gravity and rotation will be ignored here. The conservative, incompressible Boussinesq Equations with the traditional Coriolis approximation and a simple thermodynamics of density conservation are the following:

[1] $$\begin{array}{cc} \frac{Du}{D\tau }+f\hat{\zeta }\times u & =-\;\frac{1}{\rho_{0}}\nabla p+\frac{\rho }{\rho_{0}}\;\nabla \Phi \\ \frac{D\rho }{D\tau } & =0\\ \nabla \cdot u & =0\;. \end{array}$$

The (east,north, “up”) vector components are (ξ,η,ζ) and (u,v,ω). *p* is the dynamic pressure and *ρ* is the disturbance density (after subtracting off the background hydrostatic resting state); *ρ*_0_ is a reference value for total density. Gravity is implicit in the geopotential function Φ. For a uniform geopotential field, Φ=−gζ. As usual, the material derivative is

[2] $$\begin{array}{c} \frac{D\;}{\mathrm{Dt}}\;=\;\partial_{\tau }+u\partial_{\xi }+v\partial_{\eta }+\omega \partial_{\zeta }\;. \end{array}$$

Now make a transformation of Eq. **1** into nonorthogonal geopotential coordinates, (x,y,Z,t), defined by

[3] $$\begin{array}{c} x=\xi \;,\;y=\eta \;,\;Z=\;-\;\frac{\Phi }{g}\;,\;t=\tau \;. \end{array}$$

**Fig. 1.**
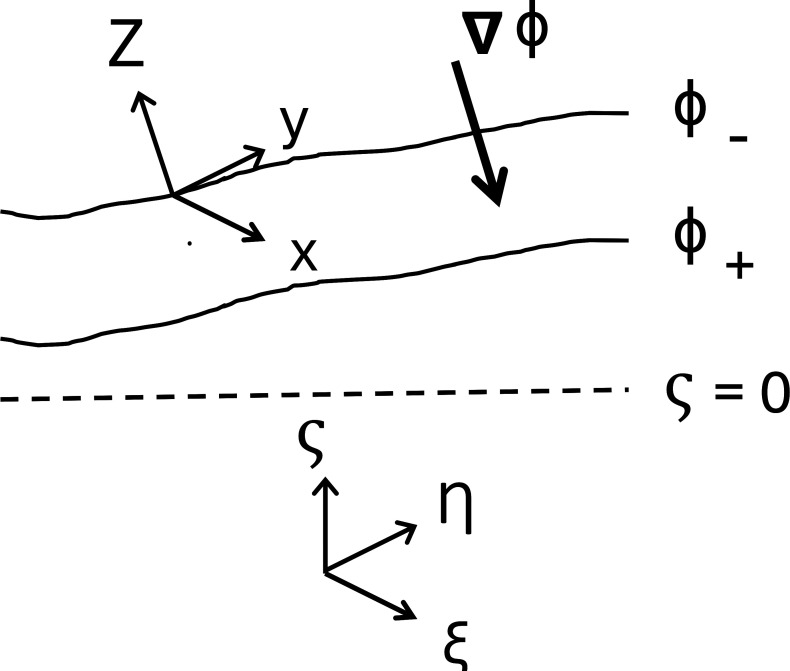
Sketch of the geometry of the absolute Cartesian coordinates (ξ,η,ζ) and the transformed geopotential coordinates (x,y,Z) in relation to the gravity-rotation potential function Φ and its gradient ∇Φ. The horizontal coordinates, (ξ,η) and (x,y), are the same in the two systems. The absolute coordinates are orthogonal, while the geopotential coordinates are not.

Thus, (east,north,time) retain their original meaning, but now “up” is perpendicular to geopotential surfaces, and *Z* is a height of and/or distance between such surfaces (Fig. 1). We assume that the domain in *Z* is of limited extent (i.e., a small fraction of solid Earth’s radius), and the local origin of *Z* is within the domain of interest. Near Earth’s surface the Jacobian of this coordinate transformation, $\partial_{\zeta }Z$, is nonzero. For definiteness, we specify that the average direction of the unit vector $\hat{Z}$ is equal to that of $\hat{\zeta }$; i.e.,

[4] $$\begin{array}{c} \hat{\zeta }=-\frac{\langle \nabla \text{Φ}\rangle }{\left|\langle \nabla \text{Φ}\rangle \right|}\equiv \frac{\langle \hat{\text{Z}}\rangle }{\left|\langle \hat{\text{Z}}\rangle \right|}, \end{array}$$

where the angle brackets denote a volume average over the domain. Similarly, the average value of the gravitational acceleration *g* is the constant,

[5] $$\begin{array}{c} g=\left|\langle \nabla \text{Φ}\rangle \right|. \end{array}$$

The basics of this representation of Earth’s gravity-rotational force are presented in ref. 11, section 1.3.2 with an opposite-sign convention for Φ but with the same net force, of course.

The mathematics of this transformation are a special case of the results in ref. 12, which deals with coordinate transformations like Eq. **3** in which only the vertical coordinate is transformed to a general s(ξ,η,ζ,τ) (e.g., pressure, entropy/density, or terrain-following coordinates); this approach was pioneered in ref. 13. The derivation details mostly will not be repeated here except to present the results; however, the special case of hydrostatic balance with a geopotential coordinate *s* = *Z* is different in some aspects from those considered in ref. 12, and its important properties are derived in Section 3.

The resulting horizontal momentum equations are

[6] $$\begin{array}{cc} \frac{\mathrm{Du}}{\mathrm{Dt}}-fv & =-\;\frac{1}{\rho_{0}}p_{x}\\ \frac{\mathrm{Dv}}{\mathrm{Dt}}+fu & =-\;\frac{1}{\rho_{0}}p_{y}\;, \end{array}$$

where (u,v) are the horizontal velocities, the traditional approximation for the Coriolis force has not been changed, and subscripts denote partial derivatives. The substantial derivative has the same meaning as Eq. **2**, but a different expression,

[7] $$\begin{array}{c} \frac{D\;}{\mathrm{Dt}}\;=\;\partial_{t}+u\partial_{x}+v\partial_{y}+w\partial_{Z}\;, \end{array}$$

and the transformed vertical velocity is

[8] $$\begin{array}{c} w\;=\;\omega Z_{\zeta }+uZ_{\xi }+vZ_{\eta }\;. \end{array}$$

The general vertical momentum equation could be obtained by projection of the first equation in Eq. **1** onto the unit vector $\hat{Z}$, with an accompanying decision about the Coriolis force approximation. However, for most large-scale oceanic and atmospheric dynamics, the hydrostatic approximation suffices as part of the Primitive Equations, a subset of Eq. **1**. In geopotential coordinates, the *Z*-momentum equation is

[9] $$\begin{array}{c} \frac{1}{\rho_{0}}p_{Z}\;=\;-\;g\frac{\rho }{\rho_{0}}\;. \end{array}$$

The remarkable result—i.e., the title of this paper—is that the horizontal pressure gradient force in Eq. **6** and the hydrostatic balance Eq. **9** have isomorphic functional forms between the cases of variable Φ with geopotential coordinates and a uniform vertically-aligned gravity field in absolute coordinates. This result is derived in Section 3.

The density conservation equation in Eq. **1** is formally unchanged,

[10] $$\begin{array}{c} \frac{D\rho }{\mathrm{Dt}}\;=\;0\;, \end{array}$$

with the transformation of the material derivative Eq. **2**. The incompressible continuity equation is

[11] $$\begin{array}{c} \partial_{x}\left[\frac{u}{Z_{\zeta }}\right]\;+\;\partial_{y}\left[\frac{v}{Z_{\zeta }}\right]\;+\;\partial_{Z}\left[\frac{w}{Z_{\zeta }}\right]\;=\;0\;. \end{array}$$

In summary, the hydrostatic Primitive Equations in geopotential coordinates are Eqs. **6**–**11**. They closely resemble the mathematical form of the Primitive Equations in a uniform gravity field in absolute coordinates, with the exception of the extra metric factor $1/Z_{\zeta }$ in the continuity Eq. **11**, as well as the implicit reinterpretations of the vertical coordinate *Z* in Eq. **3**, material derivative Eq. **2**, and vertical velocity *w* in Eq. **8**.

## 2. Discussion and Conclusions

In a coordinate system not aligned with gravity, there is indeed a nonvertical gravity force. However, as anticipated by ref. 1, this force can be accommodated in a fluid at rest in hydrostatic balance; i.e., there is a balancing nonvertical pressure-gradient force. This seems likely to be the error in ref. 4, and a similar error is made in ref. 14 and its cited literature. This misalignment is absent in geopotential coordinates, and its expression of the force balance in the background resting state is simpler.

The evident conclusion from Section 1 is that there is nothing in the transformed Primitive Equations that resembles a “horizontal gravity” force, although there is spatial variation of the gravitational-centrifugal potential field Φ that is mostly implicit. However, as remarked at the end of that section, it does appear explicitly in a few places.

For the latter it is useful to partition Φ into its average component and small deviation,

[12] $$\begin{array}{c} \Phi \;=\;-\;g\zeta +\tilde{\Phi }\left(\xi ,\eta ,\zeta \right)\;, \end{array}$$

whence

[13] $$\begin{array}{c} Z=\zeta +\tilde{Z}\left(\xi ,\eta ,\zeta \right) \end{array}$$

and

[14] $$\begin{array}{c} \tilde{Z}\;\approx \;-\;\frac{1}{g}\tilde{\Phi }\;. \end{array}$$

With this approximation, the geopotential vertical velocity in Eq. **8** becomes

[15] $$\begin{array}{c} w\;=\;\omega \;+\;\left(\;u{\tilde{Z}}_{\xi }+v{\tilde{Z}}_{\eta }+\omega {\tilde{Z}}_{\zeta }\;\right), \end{array}$$

and after multiplication by *Z*_*ζ*_ and application of the derivative transformation formulas Eq. **17**, the continuity Eq. **11** becomes

[16] $$\begin{array}{cc} & \left[\;u_{x}\;-\;\left(\frac{{\tilde{Z}}_{\xi \zeta }}{Z_{\zeta }}\;-\;\frac{{\tilde{Z}}_{\xi }{\tilde{Z}}_{\zeta \zeta }}{Z_{\zeta }^{2}}\;\right)\;u\;\right]\\ & \;+\;\left[\;v_{y}\;-\;\left(\frac{{\tilde{Z}}_{\eta \zeta }}{Z_{\zeta }}\;-\;\frac{{\tilde{Z}}_{\eta }{\tilde{Z}}_{\zeta \zeta }}{Z_{\zeta }^{2}}\;\right)\;v\;\right]\\ & \;+\;\left[\;w_{z}\;-\;\frac{{\tilde{Z}}_{\zeta \zeta }}{Z_{\zeta }^{2}}\;w\;\right]\;=\;0\;, \end{array}$$

because the leading term in Eq. **13** has no horizontal derivative nor second vertical derivative in absolute coordinates. In each square-bracketed term, the expected velocity gradient is first and the metric correction in parentheses is second. Note that the metric terms involve second derivatives of the transformed vertical coordinate *Z* (Section 3). The appearance of such metric terms in the continuity equation is usual in nonorthogonal coordinate systems with a transformed vertical coordinate (12), and they reflect the distorted geometry of the transformation. Physically restated, the simple Cartesian expression of mass conservation equal to volume conservation in the Boussinesq continuity equation in Eq. **1** has a deformed differential-geometry expression in Eq. **16**.

It would be straightforward to adapt a Cartesian Primitive Equation model code to a geopotential one by including the extra metric terms in Eq. **16** based on the measured geopotential fields, while otherwise leaving the model equations unaltered and remembering that a transformation back into an absolute vertical coordinate *ζ* and an absolute vertical velocity *ω* could be done diagnostically post hoc.

**Fig. 2.**
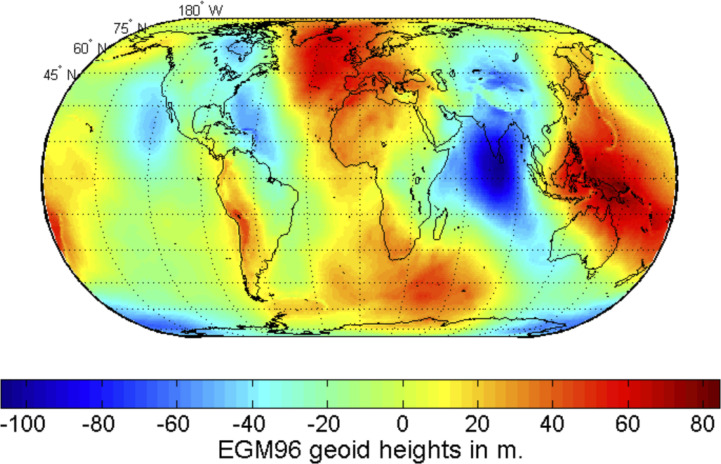
The geoid undulation, geoid height, or geoid anomaly is the height of the geoid relative to a given spheroid of reference. This plot is from EGM96 (15), but a more modern and complete dataset is EGM2008 available at https://epsg.org/crs_3855/EGM2008-height.html, with an even newer release expected soon.

There are several ways to estimate how different its answer might be. One comes from a geoid map (Fig. 2). Compared to a flat geoid surface the measured geoid has horizontal slopes on the order of ${\tilde{Z}}_{\xi }∼10^{-4}$. Another is the relative correction to vertically uniform gravity by expanding the $R^{-2}$ dependence of a spherical geopotential about the mean radius *R*_0_ with a domain height of *H*, yielding a relative correction of $2H/R_{0}∼10^{-3}$, where *H* is the domain height. Both of these estimates are comfortably small compared to other inaccuracies in geophysical fluid simulations.

Thus, there is no horizontal gravity force in geopotential coordinates, consistent with most modeling practices for large-scale circulation, albeit often implicitly so. Furthermore, the traditional neglect of variable gravity in oceanic and tropospheric simulations is a fairly safe practice, although it also could be extended by adding the metric terms indicated here in an appropriate planetary-geometry geopotential-coordinate system.

## 3. Methods

Here, we derive the pressure gradient and hydrostatic balance relations in geopotential coordinates. With the coordinate definition in Eq. **3**, first consider the transformation relationship for derivatives:

[17] $$\begin{array}{cc} \partial_{\xi } & =\;\partial_{x}+Z_{\xi }\partial_{Z}\\ \partial_{\eta } & =\;\partial_{y}+Z_{\eta }\partial_{Z}\\ \partial_{\zeta } & =\;Z_{\zeta }\partial_{Z}\\ \partial_{\tau } & =\;\partial_{t}\;. \end{array}$$

Next consider the unit vectors in the transformed coordinates:

[18] $$\begin{array}{c} \hat{x}\;=\;\hat{\xi }\;,\;\hat{y}\;=\;\hat{\eta }\;,\;\hat{Z}\;=\;-\;\frac{\nabla \Phi }{\left|\nabla \Phi \right|}\;. \end{array}$$

The gradient operator can be expressed in the orthogonal absolute coordinates as

[19] $$\begin{array}{c} \nabla \;=\;\hat{\xi }\partial_{\xi }+\hat{\eta }\partial_{\eta }+\hat{\zeta }\partial_{\zeta }\;. \end{array}$$

Thus, using this relation and the definition of *Z* in Eq. **3**, the relations in Eq. **18** can be inverted to give

[20] $$\begin{array}{c} \hat{\xi }\;=\;\hat{x}\;,\;\hat{\eta }\;=\;\hat{y}\;,\;\hat{\zeta }\;=\;\frac{1}{Z_{\zeta }}\;\left(\;|\nabla Z|\hat{Z}\;-\;Z_{\xi }\hat{x}\;-\;Z_{\eta }\hat{y}\;\right)\;. \end{array}$$

These imply the transformation rule for a gradient (applied here to the pressure *p*):

[21] $$\begin{array}{cc} \nabla p & =\;p_{\xi }\hat{\xi }+p_{\eta }\hat{\eta }+p_{\zeta }\hat{\zeta }\\ & =\;\left(\;\partial_{x}p+Z_{\xi }\partial_{Z}p\;\right)\;\hat{x}\;+\;\left(\;\partial_{y}p+Z_{\eta }\partial_{Z}p\;\right)\;\hat{y}\\ & \;\;\;+\;Z_{\zeta }\partial_{Z}p\;\frac{1}{Z_{\zeta }}\;\left(\;|\nabla Z|\hat{Z}\;-\;Z_{\xi }\hat{x}\;-\;Z_{\eta }\hat{y}\;\right)\\ & =\;p_{x}\hat{x}\;+\;p_{y}\hat{y}\;+\;\left|\nabla Z\right|p_{Z}\hat{Z}\;. \end{array}$$

Therefore, the pressure gradient force retains its same functional form in the transformed horizontal momentum Eq. **6** as in the absolute horizontal momentum equations.

From the pressure- and potential-gradient terms in Eq. **1**, the hydrostatic “vertical” momentum equation in geopotential coordinates is

[22] $$\begin{array}{cc} \hat{Z}\cdot \frac{1}{\rho_{0}}\nabla p & =\;\hat{Z}\cdot \frac{\rho }{\rho_{0}}\nabla \Phi \\ \frac{1}{\rho_{0}}\left|\nabla Z\right|p_{Z} & =\;\left(\;-\;\frac{\nabla \Phi }{\left|\nabla \Phi \right|}\;\right)\cdot \frac{\rho }{\rho_{0}}\nabla \Phi \\ \frac{1}{\rho_{0}}p_{Z} & =\;-\;g\frac{\rho }{\rho_{0}}\;, \end{array}$$

where we have made use of Eqs. **3** and **18**. This, too, has the same form as hydrostatic balance in a uniform gravity field in absolute coordinates, but now the variable gravity field is implicit in the transformed geopotential height coordinate *Z*.

Notice that a factor of |∇Z| cancels out in going from the penultimate to the final relation in Eq. **22**. This is permitted because |∇Z|≠0 for geopotential coordinates. In a generalization to nonhydrostatic dynamics for small-scale or rapidly changing flows, this factor must be retained along with the vertical acceleration, $Dw/Dt\;\hat{\zeta }$, in Eq. **1**, which leads to a more complicated vertical momentum balance than Eq. **22** in geopotential coordinates. Similarly, the complete Coriolis force could be retained. Nevertheless, as discussed in Section 2, this added complexity is mathematically well behaved because of the relative smallness of $\tilde{Z}$ and its derivatives.

## Data Availability

There are no data underlying this work.
